# Evidencia actual de la infección por SARS-COV-2 en la gestación: Revisión de alcance**

**DOI:** 10.15649/cuidarte.2265

**Published:** 2022-08-27

**Authors:** Angel Flaminio Guiza Romero, Gabriela Saldaña Agudelo, Lucy Marcela Vesga Gualdrón

**Affiliations:** 1 Enfermero, Facultad de enfermería, Universidad Nacional de Colombia. Sede Bogotá, Colombia. Email: afguizar@unal.edu.co Universidad Nacional de Colombia Facultad de enfermería Universidad Nacional de Colombia Bogotá Colombia afguizar@unal.edu.co; 2 Enfermera, Facultad de enfermería, Universidad Nacional de Colombia. Sede Bogotá, Colombia. Email: gsaldanaa@unal.edu.co Universidad Nacional de Colombia Facultad de enfermería Universidad Nacional de Colombia Bogotá Colombia gsaldanaa@unal.edu.co; 3 Profesora asistente, Departamento de enfermería, Facultad de enfermería, Universidad Nacional de Colombia. Sede Bogotá, Colombia. Email: lmvesgag@unal.edu.co Universidad Nacional de Colombia Departamento de enfermería Facultad de enfermería Universidad Nacional de Colombia Bogotá Colombia lmvesgag@unal.edu.co

**Keywords:** Coronavirus, COVID 19, SARS-CoV-2, Embarazo, Recién Nacido, Coronavirus, COVID 19, SARS-CoV-2, Pregnancy, Infant, Newborn, Coronavirus, COVID 19, SARS-CoV-2, Gravidez, Recém-Nascido

## Abstract

**Introducción:**

El SARS-CoV-2 es un Betacoronavirus, así como el SARS-CoV y el MERS-CoV, ambos asociados a abortos espontáneos, parto prematuro, morbi-mortalidad materna y alto número de ingresos a UCI en las gestantes. Además, al ser un virus nuevo, se conoce poco sobre los efectos en la gestación. Esta revisión tiene como objetivo analizar la evidencia disponible sobre el SARS-CoV-2 en la gestación.

**Materiales y métodos:**

Se realizó una búsqueda de la literatura en PubMed, ProQuest, Scopus, BVS y SciElo. Se realizó la crítica de la evidencia y la extracción de la información con dos instrumentos propuestos por el Instituto Joanna Briggs. Lo anterior bajo las directrices de PRISMA-ScR.

**Resultados:**

Se incluyeron 85 artículos que evidenciaron que la mayoría de gestantes con SARS-CoV-2 desarrollaron enfermedad leve a moderada, pero presentaron mayor riesgo de muerte y complicaciones comparado con las pacientes no embarazadas. Se documentó bajo riesgo de transmisión vertical y los resultados perinatales se asociaron a la severidad del cuadro clínico materno. La efectividad del tratamiento no fue concluyente.

**Discusión:**

Se discute la presentación clínica de la infección en las gestantes, la transmisión vertical, el tratamiento, la gravedad de la enfermedad y los desenlaces neonatales.

**Conclusiones:**

La COVID-19 en la gestación es una complicación que genera mayor morbimortalidad, por lo que es de vital importancia el desarrollo de más investigaciones que amplíen la comprensión de su comportamiento, las implicaciones fisiológicas, emocionales y el posible tratamiento. Esta revisión hace un análisis riguroso de la calidad de los estudios y aporta información valiosa de la evidencia.

## Abstract (en)

**Introduction:**

SARS-CoV-2 is a betacoronavirus as well as SARS-CoV and MERS-CoV, both associated with spontaneous miscarriage, preterm birth, maternal morbidity and mortality and a higher number of ICU admissions for pregnant women. Being a new virus, its effects on pregnancy are little known. This review aims to analyze the available evidence on SARS-CoV-2 in pregnancy.

**Materials and Methods:**

A literature review was conducted in PubMed, ProQuest, Scopus, BVS and SciElo. Evidence criticism and information extraction were conducted using two instruments from the Joanna Briggs Institute, following the PRISMA-ScR guidelines.

**Results:**

85 articles were included evidencing that most pregnant women with SARS-COV-2 suffered mild to moderate disease and were at a higher risk of death and complications compared to non-pregnant patients. Low risk of vertical transmission was documented and adverse perinatal outcomes were associated with severe maternal clinical manifestations. The effectiveness of treatment was inconclusive.

**Discussion:**

Clinical presentation of infection in pregnant women, vertical transmission, treatment, disease severity and neonatal outcomes were discussed.

**Conclusions:**

COVID-19 during pregnancy is a complication that generates greater morbidity and mortality, for which it is vital to develop further research on the understanding of the behavior, physiological and emotional implications and possible treatment. This review makes a rigorous analysis of the quality of studies and provides valuable information from evidence.

## Resumo

**Introdujo:**

O SARS-CoV-2 é um betacoronavírus, assim como o SARS-CoV e o MERS-CoV, ambos associados ao aborto espontaneo, parto prematuro, morbidade e mortalidade materna e alto número de internares na UTI em mulheres grávidas. Além disso, sendo um novo virus, pouco se sabe sobre seus efeitos na gravidez. Esta revisao tem como objetivo analisar as evidencias disponíveis sobre o SARS-CoV-2 na gravidez.

**Materiais e métodos:**

Foi realizada uma pesquisa bibliográfica na PubMed, ProQuest, Scopus, BVS e SciElo. A analise crítica das provas e a extragáo de dados foram realizadas utilizando dois instrumentos propostos pelo Instituto Joanna Briggs sob as diretrizes do PRISMA-ScR.

**Resultados:**

85 artigos foram incluidos, mostrando que a maioria das mulheres grávidas com SRA-CoV-2 desenvolveu doenga leve a moderada, mas tinha um risco maior de morte e complicares em comparagáo com pacientes náo grávidas. Baixo risco de transmissáo vertical foi documentado e os resultados perinatais foram associados a gravidade do quadro clínico materno. A eficácia do tratamento foi inconclusiva.

**Discussao:**

Apresentagáo clínica da infecgáo em mulheres grávidas, transmissáo vertical, tratamento, gravidade da doenga e resultados neonatais sáo discutidos.

**Condusdes:**

A COVID-19 na gravidez é uma complicagáo que gera maior morbidade e mortalidade, portanto, mais pesquisas para expandir a compreensáo de seu comportamento, implicares fisiológicas e emocionais, e o tratamento potencial sáo de vital importancia. Esta revisáo fornece uma análise rigorosa da qualidade dos estudos e informares valiosas a partir das evidencias.

## Introducción

El SARS-CoV-2 hace parte de los coronavirus, los cuales producen enfermedades respiratorias que van desde el resfriado común hasta la neumonía grave y SDRA. Fue notificado en diciembre del 2019 en China, y clasificado en la familia Coronaviridae tipo Beta donde se encuentran el SARS-CoV y el MERS-CoV^1^^-^^3^. La enfermedad desencadenada por este virus se denominó Enfermedad por Coronavirus del 2019 (COVID-19), de la cual alrededor del 80% de las personas se ha recuperado sin tratamiento hospitalario, siendo las personas mayores de 60 años o quienes padecen comorbilidades los más afectados. Esta enfermedad fue declarada pandemia el 11 de marzo del 2020^1^^,^^2^.

Se conoce poco sobre el comportamiento y el impacto clínico de la infección viral en la mujer gestante, siendo esto importante por los cambios inmunológicos propios de la gestación, en especial la supresión de la función de las células T, que pueden influir en la patogénesis de enfermedades infecciosas de etiología viral y generar predisposición a un curso clínico más severo por reducción de la respuesta inflamatoria, y la probabilidad de parto prematuro o aborto^4^. Adicionalmente, el SARS- CoV-2 tiene una similitud en su secuencia genética de aproximadamente el 79% con el SARS-CoV, el cual se asoció a un aumento de abortos espontáneos, parto prematuro, morbilidad materna grave y mortalidad considerable^3^^,^^5^^,^^6^. Por otro lado, presenta similitud genética del 50% con el MERS-CoV, el cual está asociado a un alto número de ingresos a unidad de cuidados intensivos (UCI), aborto espontáneo, parto prematuro y de mayores tasas de morbi-mortalidad en gestantes^3^^,^^7^^-^^9^.

**Figura 1:**
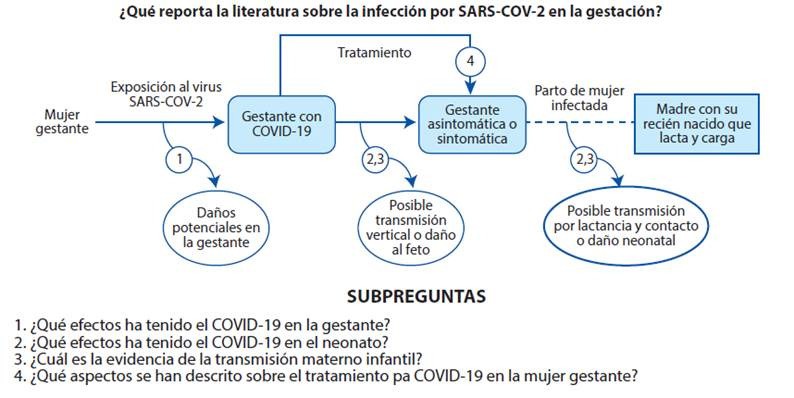
Marco analítico de la revisión de alcance del conocimiento actual sobre el SARS- COV-2 y la gestación.

Lo anterior, genera grandes incógnitas alrededor de los efectos de la COVID-19 en la gestación, por lo que se plantea la siguiente pregunta de investigación: ¿Que reporta la literatura sobre la infección por SARS-CoV-2 en la gestación? Se compone de 4 sub preguntas descritas en el marco analítico (Figura 1). El objetivo es mapear sistemáticamente la evidencia disponible sobre la enfermedad de COVID-19 en la gestación, identificando la presentación de la enfermedad en esta población, los efectos maternos y neonatales, la posibilidad de trasmisión materno infantil y el tratamiento en la gestante, mediante una revisión de alcance.

## Materiales y Métodos

### Marco de la revisión

Se realizó una búsqueda exhaustiva de la literatura el 08 de agosto del 2020 en las bases de datos PubMed, ProQuest, Scopus, BVS y SciElo. Usando las ecuaciones de búsqueda: “PREGNANCY AND COVID-19”, “SARS COV 2 AND PREGNANCY”, “PREGNANCY AND CORONAVIRUS”. Se incluyeron artículos que respondieron a las preguntas de investigación, que tenían como participantes mujeres gestantes, en posparto temprano con COVID-19 y recién nacidos en periodo neonatal precoz de madres con dicha enfermedad, de tipo experimental, observacional descriptivo o analitico, revisiones sistematica y artículos de opinión, con acceso a texto completo, publicados ono y desarrollados durante los años 2019 o 2020. Solo se incluyeron artículos en idioma inglés, portugués y español; se excluyeron los demás idiomas por la dificultad de comprensión y posible sesgo en el análisis de los datos. Adicional a lo anterior, se realizó una búsqueda secundaria en las referencias de los artículos encontrados. El protocolo de este estudio sigue los lineamientos de PRISMA-ScR^10^ y el Instituto Joanna Briggs^11^, se encuentra publicado y registrado en Open Science Framework (DOI: 10.17605/OSF.IO/JM2KX).

### Selección y recolección de la información

Después de realizada la búsqueda se eliminaron los artículos duplicados y aquellos que no cumplían con los criterios de inclusión. Posterior a esto, se realizó la crítica de la evidencia con las listas de chequeo propuestas por el Instituto Joanna Briggs^12^ para la evaluación crítica de la evidencia, por dos autores de manera independiente (GS y AG); las discrepancias fueron solucionadas por el tercer autor (LV), quien tenía mayor experiencia investigativa. Para esta revisión se incluyeron los artículos que cumplían con mínimo el 75% de los ítems que evaluaban la herramienta.

Para la extracción de la información se utilizó un instrumento propuesto por el Instituto Joanna Briggs^11^ para las revisiones de alcance que fue adaptado a los objetivos de la revisión. Previo a esto, se realizó una prueba piloto del instrumento para evaluar su utilidad, con 4 artículos de diferente diseño conducida por los autores de manera individual. Esta herramienta permitió detallar datos de la fuente, sus características y resultados (la información adicional se encuentra en el protocolo de investigación).

### Análisis y presentación de la información

El análisis de la información se realizó mediante tres estrategias: la primera, por medio de la tabulación de la caracterización de los estudios incluidos, la segunda se realizó empleando el software RevMan 5^13^ para la construcción de gráficas de riesgo de sesgos de los artículos, siendo riesgo alto cuando no cumplía el criterio evaluado, riesgo medio si no era claro o bajo riesgo al cumplirse. La tercera se hizo mediante un análisis de la información abarcada por cada pregunta, dando un panorama de lo que se conoce de la COVID-19 en la gestación.

## Resultados

### Selección y caracterización de los estudios

**Figura 2:**
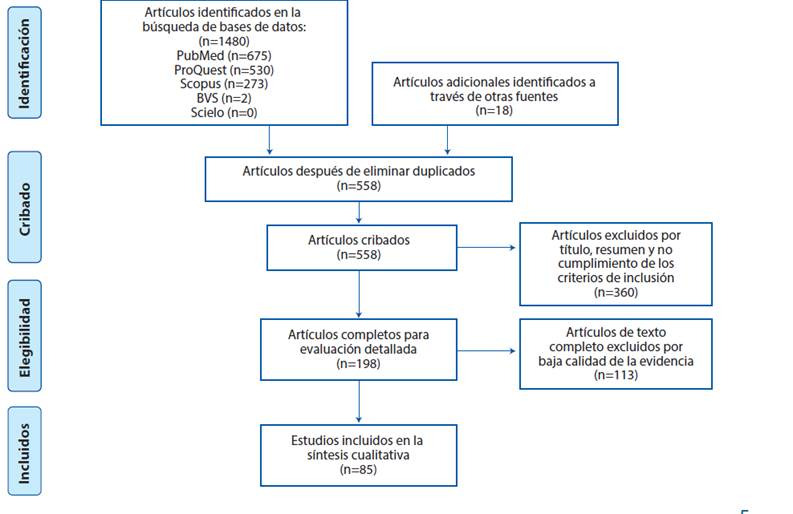
Diagrama de flujo del proceso de selección de estudios, según las directrices PRISMA-ScR^10^

A partir de la búsqueda primaria se obtuvieron 1480 artículos y 18 de la búsqueda secundaria. Después de aplicar los criterios de inclusión y exclusión, se evaluó la calidad de la evidencia de 198 artículos, obteniendo un número final de 85 artículos a incluir (Figura 2). Dentro de los artículos excluidos 326 no cumplían con el objetivo, 27 no cumplían con los diseños a incluir, 2 hablaban de otros coronavirus, 4 estaban en chino, 2 en francés y 1 en alemán. Dentro de los artículos incluidos 32 fueron opiniones clínicas, de las cuales se incluyeron comunicaciones de expertos, cartas al editor y editoriales; 35 artículos observacionales descriptivos y 13 observacionales analíticos; respecto a los artículos de revisión sólo se incluyeron revisiones sistemáticas y de alcance por la posibilidad de evaluar la calidad de la evidencia, con 5 estudios incluidos (Tabla 1).

**Tabla 1:** Caracterización de los estudios incluidos.

Características		
2019		(n)*
Fecha		(n=0)
2020		
Opiniones clínicas y comunicaciones Reportes de casos		(n=85)
		(n=21)
Tipo de Series de casos		(n=14)
^(artículo)^Estudios de cohorte		(n=7)
Estudios de casos y controles		(n=3)
Estudios transversales		(n=3)
Revisiones sistemáticas		(n=5)
China		(n=29)
Estados Unidos		(n=12)
Españ a		(n=5)
Italia		(n=4)
Reino Unido		(n=4)
Francia		(n=3)
Canadá		(n=2)
Irán		(n=2)
Portugal		(n=2)
Rumania		(n=2)
Suecia		(n=2)
Turquía		(n=2)
Australia		(n=1)
País		
Brasil		(n=1)
Corea del Sur		(n=1)
Egipto		(n=1)
India		(n=1)
Irlanda		(n=1)
Países Bajos		(n=1)
Perú		(n=1)
Rusia		(n=1)
Singapur		(n=1)
Suiza		(n=1)
Varios países		(n=5)
Inglés		(n=83)
Idioma Español		(n=2)
Portugués		(n=0)
Artículos publicados		(n=84)
Artículos no publicados		(n=1)
Pregunta que responde	(n)*	Referencia
¿Qué efectos ha tenido el	(n=73)	14-16,18-33,35-63,65-70,72,73,75,77-84,86-92,94,95,97
COVID-19 en la gestante?		
¿Qué efectos ha tenido el	(n=57)	14,15,17,19,24,27,29,32,35,38,41,44,46,48,49,51,53,56,58,60,61,64,66,67,69,
COVID-19 en el neonato?		70,72,73,75,80,83,84,86,87,90-92,95,97,98
¿Cuál es la evidencia de la	(n=56)	14,17,19,21,24,27,29,32,34,35,39,41,44,46,48,49,51,54,57,59,63,67,69,80,84,87,
transmisión materno infantil?		90,93,95,98
¿Qué aspectos se han descrito	(n=43)	14-16,19,24,25,32,33,38,40,44,45,48-51,53-59,61,65,67,70,72,73,75,77-83,86,87,
sobre el tratamiento para		90,91,95,97
COVID-19 en la gestante?		

* n= número de artículos

### Calidad de la evidencia

**Figura 3:**
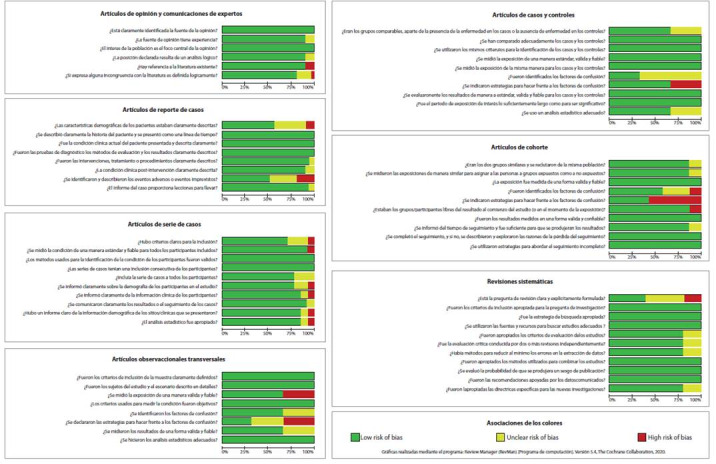
Riesgo de sesgos por diseño

El 15% de los estudios de opinión no hicieron suficiente referencia a la literatura existente y el 20% no definió lógicamente las incongruencias expresadas con la literatura. Adicionalmente, el 40% de los reportes de caso no describieron claramente las características demográficas de las participantes y el 20% no identificó eventos adversos. La variable que más presentó algún porcentaje de alto riesgo de sesgo en los estudios observacionales analíticos, fue la ausencia de estrategias para hacer frente a los factores de confusión. Por otro lado, las series de casos obtuvieron más preguntas con alto riesgo de sesgo (6 de 10 preguntas), mientras que las revisiones sistemáticas sólo presentaron algún porcentaje de alto riesgo de sesgo al no expresar claramente la pregunta de investigación (Figura 3).

### Reporte de la literatura sobre el SARS-CoV-2 en la gestación

A continuación, se describe lo reportado en la literatura por cada sub pregunta planteada:

### Efectos en la gestante

Características clínicas: En 41 artículos se describieron los síntomas de COVID-19 de 627 gestantes o en puerperio inmediato y mediato, de las cuales el 18,7% (n=117) fueron asintomáticas. En las gestantes sintomáticas, los síntomas más comunes fueron fiebre (47,4%, n=297), tos (42,1%, n=263) y dificultad para respirar o disnea (17,2%, n=108). Otros síntomas descritos fueron fatiga (6,5%, n=41), mialgia (8%, n=50), dolor de garganta (6,4%, n=40), síntomas gastrointestinales como vómito y diarrea (4,1%, n=26), congestión nasal (4,3%, n=27), cefalea (3,7%, n=23) y pérdida del gusto o el olfato (3,2%, n=20). Los síntomas que se describieron en muy poca proporción (0,2% - 2,2%) fueron malestar general, dolor torácico, rash cutáneo y tos con expectoración^14^^,^^15^^,^^19^^,^^20^^,^^22^^-^^24^^,^^27^^,^^29^^,^^30^^,^^32^^,^^35^^,^^38^^,^^39^^-^^42^^,^^45^^-^^48^^,^^52^^,^^53^^,^^56^^-^^58^^,^^61^^,^^62^^,^^67^^,^^72^^,^^73^^,^^78^^-^^80^^,^^83^^,^^84^^,^^87^^,^^90^^,^^91^^,^^95^^,^^97^^).^ Un estudio de casos y controles^77^, describió que el dolor abdominal fue un síntoma exclusivo de las gestantes, que no fue descrito en mujeres no embarazadas.

Tres estudios con pruebas universales de SARS-CoV-2 en gestantes encontraron mayor porcentaje de pacientes asintomáticas, dos lo describieron en el 60%^21^ y 73,3%^36^ de la muestra Mientras que el tercero, reportó que el 97% de las gestantes con COVID-19 fueron asintomáticas o presentaron síntomas leves^70^.

Se informaron los resultados en la tomografía axial computarizada (TAC) de tórax de 218 gestantes con COVID-19 en 23 estudios^14^^,^^22^^-^^24^^,^^29^^,^^32^^,^^41^^,^^42^^,^^45^^,^^46^^,^^52^^,^^61^^,^^67^^,^^72^^,^^77^^,^^80^^,^^82^^,^^83^^,^^86^^,^^87^^,^^90^^,^^91^^,^^97^_,_ de las cuales el 91,3% (n=199) presentaron sombras irregulares en vidrio esmerilado, el 6,4% (n=14) reportaron consolidaciones, solo el 0,5% (n=1) tenían otras alteraciones como infiltrados pulmonares, derrame pleural o atelectasias y el 3,7% (n=8) no presentaron alteraciones.

También se documentaron los resultados en la radiografía (Rx) de tórax en 13 gestantes con COVID-19 en ocho estudios^15^^,^^27^^,^^38^^,^^39^^,^^46^^,^^52^^,^^80^^,^^97^, de ellas 53,8% (n=7) desarrollaron consolidaciones pulmonares, el 30,8% (n=4) infiltrados pulmonares y el 15,4% (n=2) patrón en vidrio esmerilado y atelectasias cada uno. 1 presentó derrame pleural, 1 patrón intersticial-alveolar y 1 no tuvo alteraciones en la radiografía. Se encontró que más de la mitad de las mujeres tuvo compromiso pulmonar bilateral difuso ^14^^,^^15^^,^^24^^,^^27^^,^^29^^,^^32^^,^^38^^,^^42^^,^^45^^,^^46^^,^^52^^,^^61^^,^^67^^,^^72^^,^^80^^,^^82^^,^^83^^,^^86^^,^^87^^,^^91^^,^^97^_._

Sobre las alteraciones en los laboratorios, se hallaron informes de 389 gestantes o en puerperio mediato con COVID-19 en 30 artículos ^14^^,^^15^^,^^20^^,^^22^^,^^23^^,^^24^^,^^32^^,^^35^^,^^38^^,^^39^^,^^40^^,^^41^^,^^46^^,^^48^^,^^51^^,^^52^^,^^56^^,^^58^^,^^61^^,^^67^^,^^79^^,^^80^^,^^83^^)-(^^87^^,^^90^^,^^91^^,^^95^^,^^97^_,_ donde las alteraciones en los laboratorios mayoritariamente descritas fueron la elevación de la proteína C reactiva (PCR), linfocitopenia y leucocitosis o leucopenia. Pocos estudios de los incluidos en esta lista consideraron otros marcadores como el LDH y ferritina.

Pereira, et al^73^ y Yang, et al^90^ estuvieron de acuerdo en que las pacientes con enfermedad grave presentan anomalías más prominentes en los laboratorios, los primeros encontraron asociación entre los niveles elevados de PCR, Dímero D y neutrófilos con la neumonía grave. Mientras que los segundos mencionan anomalías como linfocitopenia, leucopenia y trombocitopenia. Además, destacan que estas alteraciones fueron más frecuentes en el tercer trimestre del embarazo que en el primer trimestre de este. Adicionalmente, un estudio de casos y controles^77^ encontró que hubo más leucocitosis y elevación de la PCR en gestantes con COVID-19, que en mujeres no embarazadas.

Se recomendó controlar la gasometría arterial, el lactato, la función renal, hepática y las enzimas cardíacas, según lo indique la situación clínica de la gestante con SARS-CoV-2, teniendo en cuenta que se han descrito casos de choque séptico, lesión renal aguda y lesión cardíaca inducida por el virus. Así mismo, pruebas de coagulación por reporte de eventos tromboembólicos en pacientes no embarazadas con la enfermedad, lo que es importante porque las gestantes presentan un estado protrombótico y por ende mayor riesgo^28^^,^^55^.

Gravedad de la enfermedad: En 8 artículos se describió la gravedad de la enfermedad de COVID-19 en 437 gestantes o en puerperio mediato^21^^,^^22^^,^^41^^,^^58^^,^^68^^,^^70^^,^^78^^,^^90^, de las cuales el 92,2% (n=403) desarrollaron enfermedad leve a moderada y el 7,8% (n=34) enfermedad grave a crítica. Adicionalmente, un estudio de casos y controles^77^ que comparó gestantes con COVID-19 y mujeres no embarazadas, no encontró asociación entre el embarazo y la gravedad de la enfermedad o el tiempo de eliminación del virus. Es de resaltar que la gravedad de la COVID-19 en las gestantes se asoció a factores de riesgo como el sobrepeso y la obesidad pregestacional, diabetes gestacional, asma, hipertensión arterial, mayor edad materna y etapa final del embarazo^26^^,^^36^^,^^58^^,^^68^^,^^75^^,^^79^.

En 35 estudios ^14^^,^^15^^,^^19^^,^^22^^,^^23^^,^^27^^,^^31^^,^^35^^,^^36^^,^^38^^-^^40^^,^^45^^,^^46^^,^^49^^,^^53^^,^^56^^,^^58^^,^^60^^,^^62^^,^^67^^,^^68^^,^^72^^,^^73^^,^^75^^,^^78^^-^^80^^,^^82^^,^^86^^,^^87^^,^^90^^,^^91^^,^^95^^,^^96^ hubo 1601 gestantes o en posparto precoz con infección por SARS-CoV-2, de las cuales el 82,7% (n=1324) no requirieron oxígeno suplementario o ingreso a UCI, el 9,1% (n=145) recibió oxígeno suplementario, principalmente con cánula nasal. El 7,4% (n=119) ingresó a UCI, pero 47 recibieron ventilación mecánica (VM). Solo se reportaron 8 casos de oxigenación por membrana extracorpórea, 6 de terapia prono y 2 de diálisis.

Se reportó que el 1,1% (n=5) de 435 gestantes con COVID-19 presentaron aborto espontáneo^49^^,^^90^, y 17,6% (n=12) de 68 rotura prematura de membranas^57^^,^^90^^,^^91^; no está claro si dichas alteraciones se asociaron con la infección por SARS-CoV-2. Adicionalmente, 3 estudios describieron que 5,9% (n=9) de 152 gestantes con COVID-19 desarrollaron preeclampsia, 7 de ellas con enfermedad grave o crítica^58^^,^^68^^,^^75^.

Tres estudios notificaron mortalidad materna. El primero^97^ reportó el caso de una mujer con antecedentes de hipotiroidismo, quien empeoró progresivamente y falleció. El segundo^49^encontró una tasa de mortalidad materna asociada al SARS-CoV-2 de 5,8 (1,9-13,5) por 100.000 embarazos. Y el tercero^70^, encontró que la muerte materna en pacientes COVID-19 positivo (2,12%) fue ligeramente mayor al de las mujeres embarazadas COVID-19 negativo (0,95%).

Vía de parto y prematuridad : Se describió el modo de parto de 875 gestantes con COVID-19, de las cuales el 64,5% (n=564) dieron a luz por cesárea, y el 35,4% (n=310) por parto vaginal ^14^^,^^19^^,^^22^^-^^24^^,^^26^^,^^27^^,^^29^^,^^30^^,^^32^^,^^35^^,^^38^^,^^39^^,^^41^^,^^45^^,^^46^^,^^48^^,^^49^^,^^51^^-^^53^^,^^56^^-^^58^^,^^60^^-^^62^^,^^67^^,^^68^^,^^70^^,^^72^^,^^73^^,^^78^^-^^80^^,^^82^^,^^84^^,^^86^^,^^87^^,^^90^^,^^91^^,^^95^^,^^97^_;_ lo anterior puede verse influenciado por la incertidumbre ante la transmisión vertical del virus, que llevó a que esta enfermedad sea indicación de cesárea en China^59^^,^^69^. Además, un estudio de cohorte^75^ encontró que las mujeres con neumonía crítica presentaron una mayor incidencia de parto por cesárea, que aquellas con neumonía grave. Es probable que la razón de esta mayor incidencia de parto por cesárea en pacientes gestantes con neumonía grave y SDRA pulmonar, sea la dificultad para lograr las metas de oxigenación y ventilación necesarias para la unidad fetoplacentaria con el soporte ventilatorio mecánico convencional.

Por otro lado, de 792 mujeres infectadas con SARS-CoV-2, el 78,2% (n=619) presentaron parto a término y el 21,8% (n=173) parto pretérmi- no ^14^^,^^22^^-^^24^^,^^27^^,^^29^^,^^30^^,^^32^^,^^35^^,^^38^^,^^39^^,^^41^^,^^45^^,^^46^^,^^48^^,^^49^^,^^51^^-^^53^^,^^56^^-^^62^^,^^67^^,^^68^^,^^72^^,^^73^^,^^77^^-^^80^^,^^84^^,^^86^^,^^87^^,^^90^^,^^91^^,^^95^^,^^97^.

Además, se encontró mayor proporción de parto pretérmino en gestantes con COVID-19, sinto máticas y con enfermedad crítica^53^^,^^59^^,^^75^.

Salud mental: Se describieron efectos en la salud mental de las gestantes por la pandemia de COVID-19 como sufrimiento prenatal, síntomas más graves de depresión y ansiedad, preocupación por la seguridad de la lactancia materna, la vía de parto y posibles defectos de nacimiento^18^^,^^94^. Además, un estudio reportó un aumento de los casos de depresión conforme avanzaba la pandemia^89^, y Qiancheng, et al^77^, notificaron 4 embarazadas que interrumpieron el embarazo debido a preocupaciones sobre los efectos secundarios de los medicamentos, el examen radiológico y la COVID-19.

Adicional a lo anterior, se encontró que las gestantes se ven enfrentadas a situaciones estresantes como la limitación del acompañamiento durante el trabajo de parto, o la separación de sus hijos, lo cual afecta el vínculo y aumenta el riesgo de emociones negativas como mal humor, irritabilidad, culpa, entre otras. Lo que es importante ya que el estrés materno puede conducir a resultados adversos del embarazo, como parto prematuro, bajo peso al nacer, y mayor riesgo de presentar depresión posparto^42^^,^^43^.

### Efectos en el neonato

Para responder a los posibles efectos de la COVID-19 en el neonato, 57/85 artículos respondieron al interrogante, con un reporte total de 936 neonatos hijos de madres infectadas.

Características clínicas neonatales: En 33 artículos se reportó el Apgar neonatal ^14^^,^^19^^,^^21^^-^^24^^,^^27^^,^^29^^,^^30^^,^^32^^,^^35^^,^^39^^,^^45^^,^^46^^,^^48^^,^^51^^,^^52^^,^^56^^,^^57^^,^^60^^-^^62^^,^^67^^,^^72^^,^^76^^,^^78^^,^^84^^,^^86^^,^^87^^,^^90^^,^^91^^,^^95^^,^^97^^).^ El valor al minuto se notificó en *214* neonatos, de ellos el 4,2% (n=9) tuvo puntaje <5, el 1,4% (n=3) puntaje de 6 y el 94,4% (n=202) fue >7. El valor a los 5 minutos se reportó en 269 neonatos, de ellos el 3,7% (n=10) tuvo puntajes <7 y 96,3% (n=259) puntajes >7. Los puntajes bajos se asociaron a prematuridad y un caso a muerte neonatal^90^. Adicionalmente, dos estudios^53^^,^^70^ que compararon gestantes con y sin COVID-19, demostraron que no hubo diferencia en los puntajes de Apgar. Otro estudio de cohorte^79^ evidenció que la media de los puntajes de Apgar a los 5 minutos fue de 10, incluso en enfermedad materna grave.

Todos los casos de bajo peso al nacer fueron asociados a prematuridad. Se reportó el peso de 357 neonatos, de los cuales el 0,6% (n=2) registró peso <1000 g^35^^,^^67^, el 1,7% (n=6) registró peso entre 1000 g y 1500 g^27^^,^^35^^,^^39^^,^^67^, el 5% (n=18) registró peso entre 1500 g y 2500 g^17^^,^^30^^,^^35^^,^^48^^,^^78^^,^^87^^,^^91^^,^^97^, y el 92,7% (n=331) registró peso mayor a 2500 g^14^^,^^21^^,^^23^^,^^24^^,^^29^^,^^30^^,^^32^^,^^35^^,^^39^^,^^45^^,^^48^^,^^52^^,^^60^^-^^62^^,^^70^^,^^72^^,^^76^^-^^78^^,^^84^^,^^86^^,^^91^^,^^95^_._ Sumado a lo anterior, un estudio de cohorte^75^ demostró que el peso medio al nacer fue significativamente menor en el grupo de gestantes con enfermedad crítica que en el grupo con enfermedad grave, principalmente por menor edad gestacional. El uso de corticosteroides para maduración pulmonar se reportó en 75 casos^14^^,^^20^^,^^27^^,^^38^^,^^40^^,^^49^^,^^67^^,^^72^^,^^80^^,^^90^_._

De 175 neonatos, 160 no desarrollaron síntomas^29^^,^^35^^,^^41^^,^^48^^,^^60^^,^^61^^,^^67^^,^^76^^,^^78^^,^^79^^,^^83^^,^^95^^,^^97^^,^^98^ y 15 tuvieron síntomas como dificultad respiratoria (4%, n=7), gastrointestinales (2,3%, n=4), fiebre (2,3%, n=4), tos (1,1%, n=2) y taquipnea (0,6%, n=1); el 4% (n=7) desarrolló neumonía^14^^,^^32^^,^^35^^,^^39^^,^^48^^,^^72^^,^^73^^,^^95^^,^^98^. De los neonatos que presentaron síntomas, 3 eran positivos para COVID-19; en 11 neonatos positivos no se reportaron síntomas.

En relación a los laboratorios clínicos, no se reportaron anormalidades en 27/33 neonatos^14^^,^^41^^,^^57^. De los neonatos restantes, 3 presentaron linfocitopenia, de ellos uno era positivo para SARS- COV-2 y tuvo aumento de creatininfosfoquinasa, bilirrubina indirecta y alteración en pruebas de función hepática; otros hallazgos fueron leucocitosis (n=2), aumento de citoquinas (n=1) y un caso de trombocitopenia en un neonato cuya madre tenía trombocitopenia inmune^29^^,^^32^^,^^72^^,^^83^^,^^86^.

Se reportaron los hallazgos imagenológicos de 47 neonatos, 40 Rx de tórax^14^^,^^32^^,^^41^^,^^91^^,^^95^, 6 TAC^29^^,^^61^^,^^98^ y un neonato con ambas pruebas; este último era positivo para COVID-19 y presentó anormalidades en ambas^86^. En el 80,9% (n=38) de los casos no hubo anormalidades incluyendo a 2 neonatos positivos para COVID-19. Se encontraron cambios inflamatorios, infecciosos o nebulosidades difusas en el 12,8% (n=6) de las Rx de tórax, y marcaciones nodulares en el 8,5% (n=4) de las TAC.

Requerimiento de UCI: El traslado a UCI se reportó en 175 neonatos (18,7% en relación a la muestra total)^27^^,^^35^^,^^39^^,^^46^^,^^49^^,^^52^^,^^57^^,^^67^^,^^70^^,^^72^^,^^73^^,^^75^^,^^78^^,^^79^^,^^84^, asociado a prematuridad o prevención para seguimiento, aislamiento y un caso por COVID-19^49^. La necesidad de VM se reportó en 4 casos asociados a prematuridad^27^^,^^39^^,^^67^^,^^72^. Un estudio reportó el ingreso a UCI del 40% de los hijos de madres con enfermedad grave y el 83% en los casos de enfermedad crítica^75^.

Morbimortalidad neonatal: Se notificaron 10 casos de muerte perinatal (1,1%), 6 neonatos^49^^,^^70^^,^^90^y 4 mortinatos^49^^,^^58^, ninguno claramente asociado a COVID-19; 1 de ellos por estado materno grave^49^^,^^90^. Un estudio reportó mayor mortalidad neonatal en hijos de mujeres sin COVID-19 que con la enfermedad, 3,8% y 2,2%, respectivamente^70^. Las complicaciones neonatales descritas fueron 8 casos de sufrimiento fetal, 1 de asfixia neonatal grave y 1 de hemorragia alveolar; todos relacionados a prematuridad o enfermedad materna grave^53^^,^^67^^,^^72^^,^^78^^,^^91^. Un neonato tuvo sintomatología neurológica pero fue negativo para SARS-COV-2 en líquido cefalorraquídeo^84^.

### Transmisión materno-infantil

Se reportaron los resultados de pruebas de RT-PCR para SARS-COV-2 mediante hisopado naso u orofaríngeo de 887 neonatos, de ellos el 95,9% (n=851) fueron negativos ^19^^,^^21^^-^^24^^,^^57^^,^^29^^,^^30^^,^^32^^,^^35^^,^^39^^,^^41^^,^^45^^,^^46^^,^^48^^,^^49^^,^^51^^-^^54^^,^^57^^,^^59^^-^^62^^,^^67^^,^^70^^-^^73^^,^^75^^-^^80^^,^^87^^,^^91^^,^^95^^,^^97^^,^^98^_._ En un neonato negativo se detectó IgG e IgM para SARS-COV-2^29^, en otro neonato se detectó IgG en sangre de cordón umbilical, pero no IgM^76^. Un estudio detectó IgG e IgM para SARS-COV-2 en 1/23 neonato a pesar de ser negativo en RT-PCR^91^.

El 4,1% (n=36) de los neonatos resultaron positivos para SARS-COV-2 en RT- _PCR_^14^^,^^35^^,^^39^^,^^41^^,^^48^^,^^49^^,^^67^^,^^70^^,^^75^^,^^78^^,^^79^^,^^84^^,^^86^^,^^95^^,^^97^^,^^98^_,_ 3 a las 24 horas, pero negativos a los 5 días^70^_._ Otros 3 neonatos con resultados positivos en RT-PCR nasofaríngeo, tuvieron resultados negativos en pruebas de otros tejidos^27^^,^^78^^,^^86^^,^^95^. Mientras que 2 neonatos positivos tuvieron resultados positivos en otros tejidos^84^^,^^97^. Un neonato positivo reportó títulos de IgG e IgM negativos para SARS-COV-2^14^.

Se reportaron 27 pruebas de tejidos placentarios, el 85,2% (n=23) fueron negativos^32^^,^^39^^,^^45^^,^^51^^,^^52^^,^^54^^,^^71^^,^^73^^,^^86^^,^^87^^,^^91^ y en el 14,8% (n=4) positivos. Una de las muestras positivas fue de un neonato positivo en RT-PCR^84^. Otro estudio reportó 3/11 muestras de tejido placentario positivas, dos de gestantes con enfermedad crítica y una severa; los 3 neonatos resultaron negativos en RT-PCR^71^. Igualmente, se encontraron 36 análisis de SARS-COV-2 en líquido amniótico, el 94,4% (n=34) fueron negativos^22^^,^^32^^,^^39^^,^^41^^,^^51^^,^^52^^,^^54^^,^^72^^,^^80^^,^^87^^,^^90^^,^^96^, dos de ellos mediante amniocentesis en el primer trimestre del embarazo^96^; el 5,6% (n=2) de las muestras fueron positivas^84^^,^^97^. En relación al análisis de sangre en cordón umbilical, 19/20 resultaron negativos^32^^,^^45^^,^^52^^,^^54^^,^^76^^,^^86^^,^^87^^,^^90^^,^^95^, solo 1/20 fue positivo que también lo fue en líquido amniótico^97^.

Se notificaron 12/12 muestras de fluido vaginal negativas^29^^,^^32^^,^^72^^,^^76^^,^^90^^,^^97^. En una madre se reportó positividad en frotis anal y heces cuyo hijo fue negativo mediante RT-PCR^76^. Se obtuvieron 22/22 reportes negativos para SARS-COV-2 en heces y orina neonatal^51^^,^^54^^,^^57^^,^^61^^,^^72^. A cuatro neonatos se le practicaron frotis anales, dos resultaron negativos^69^^,^^84^ y dos positivos para SARS-COV-2^98^. Se informaron 20/20 análisis negativos en líquido gástrico neonatal^57^^,^^87^. Un neonato tuvo resultado positivo para COVID-19 mediante lavado broncoalveolar^84^. Se reportaron 37/37 muestras de leche materna negativas^22^^,^^29^^,^^32^^,^^45^^,^^51^^,^^54^^,^^57^^,^^72^^,^^74^^,^^76^^,^^90^_._

### Tratamiento

Se describió el tratamiento de la infección por SARS-CoV-2 de 1004 gestantes. Se reportó la administración de hidroxicloroquina en el 12,2% (n=122) de los casos^14^^,^^15^^,^^19^^,^^40^^,^^45^^,^^58^^,^^59^^,^^73^^,^^75^^,^^79^^,^^80^^,^^97^, en uno de ellos se suspendió por alteraciones cardíacas^15^; se usó cloroquina en un caso^38^. Adicionalmente, un estudio de cohorte refirió que se administró antibióticos junto con hidroxicloroquina a las mujeres sintomáticas^70^.

Se administró antibióticos en 371 gestantes^14^^,^^15^^,^^19^^,^^24^^,^^32^^,^^38^^,^^45^^,^^48^^,^^53^^,^^56^^,^^59^^,^^67^^,^^72^^,^^73^^,^^75^^-^^80^^,^^82^^,^^87^^,^^90^^,^^91^^,^^95^^,^^97^_,_ en su mayoría azitromicina y cefalosporinas para tratar la neumonía o de manera profiláctica. Una revisión sistemática^44^ reportó la terapia antibiótica en un 70,7% de los casos, por lo que se evidencia un amplio uso de estos medicamentos debido al riesgo de neumonía bacteriana secundaria al daño pulmonar causado por el virus. Sin embargo, los antibióticos deben administrarse con precaución y sólo si hay sospecha clínica de infección bacteriana^55^.

Se reportó el uso de antivirales en un 26,6% (n=267), principalmente oseltamivir, remdesivir, lopinavir/ritonavir, arbidol y ribavirina e interferón^14^^,^^15^^,^^24^^,^^32^^,^^38^^,^^45^^,^^48^^,^^49^^,^^51^^,^^53^^,^^54^^,^^56^^-^^58^^,^^61^^,^^72^^-^^75^^,^^77^^-^^80^^,^^82^^,^^86^^,^^87^^,^^90^^,^^91^^,^^95^^,^^97^_._ Un artículo sugiere que los antivirales de amplio espectro e interferón pueden ser efectivos contra la COVID-19 según lo observado durante el MER-CoV^25^, otro refiere que antivirales como ribavirina, interferón y lopinavir no fueron concluyentes según lo observado durante el SARS- CoV^16^.

El uso de corticosteroides se notificó en un 8% (n=80)^15^^,^^32^^,^^40^^,^^54^^,^^75^^,^^77^^,^^78^^,^^86^^,^^90^^,^^91^^,^^95^^,^^97^, sin incluir los usados para maduración pulmonar. Estos medicamentos deben usarse con precaución por la disminución de la respuesta inflamatoria y el aclaramiento viral, por lo que deben administrarse sólo cuando los beneficios superen los riesgos^81^. La tromboprofilaxis farmacológica se reportó en 82 casos con heparina de bajo peso molecular^15^^,^^42^^,^^73^^,^^75^^,^^79^^,^^80^. Los medicamentos que alteren la coagulación deben usarse con precaución por el riesgo de trombocitopenia en algunos pacientes con COVID-19, incluyendo una evaluación exhaustiva a quienes se les prescriba aspirina por riesgo de preeclampsia^65^.

Se encontraron otros tratamientos como medicinas tradicionales chinas como Lianhua qingwen y Jinye Baidu en 36 casos^32^^,^^48^^,^^78^^,^^95^, la transfusión de plasma de convalecientes de COVID-19 en tres gestantes^15^^,^^75^^,^^87^ y la administración de inmunoglobulinas^77^^,^^83^.

## Discusión

Esta revisión de alcance, tuvo un gran número de artículos, que permitió abarcar un amplio número de gestantes y neonatos, además de reportar los efectos de la COVID-19 no solo a nivel físico, sino también a nivel mental, proporcionando una mirada más amplia. Así mismo, se encontró el reporte de una gran variedad de muestras de tejidos, que permitieron comprender mejor la posibilidad de transmisión materno infantil, aspecto que no se evidenció en revisiones pasadas porque no contaban con la misma cantidad o tipos de tejidos. Adicionalmente, es de destacar que solo se incluyeron estudios que alcanzaron un alto nivel de calidad, después de realizar la crítica de la evidencia.

No obstante, la revisión cuenta con algunas limitaciones como la concentración de estudios provenientes de China o Estados Unidos, el poco reporte de eventos adversos o de factores de confusión, la novedad del virus y la poca cantidad de estudios analíticos junto con la ausencia de estudios experimentales. Así mismo, se encontró que la mayoría de los estudios incluidos sólo reportaban los desenlaces de mujeres con claros síntomas de la enfermedad y fueron muy pocos los casos donde se tomaron pruebas universales para SARS-CoV-2, lo que puede sesgar la información recogida.

Los resultados de la presente revisión concuerdan con lo reportado en cinco revisiones sistemáticas, donde tres de ellas encontraron que los síntomas más recurrentes entre las gestantes con COVID-19 eran la fiebre y la tos^31^^,^^44^^,^^92^; y dos encontraron que la mayoría de gestantes infectadas presentan alteraciones imagenológicas como sombras irregulares u opacidad en vidrio esmerilado^45^^,^^69^. De la misma manera, tres revisiones^31^^,^^44^^,^^69^ describen la elevación de la PCR y la linfopenia como unas de las alteraciones más frecuentes en embarazadas con COVID-19. Además, una de ellas^31^ describe un aumento de Dímero D en el 22,3% de 385 gestantes infectadas.

Dos revisiones encontraron una proporción de gestantes con COVID-19 grave o crítico <8%, siendo coherente con los resultados expresados en esta revisión^31^^,^^92^. Sin embargo, otros estudios^47^^,^^88^ proponen pruebas universales para SARS-CoV-2 a las gestantes como alternativa para determinar el impacto real de la COVID-19 en el embarazo. Teniendo en cuenta que la gravedad del virus no se puede analizar sin datos poblacionales a gran escala de varios países, con el ajuste de los factores de confusión.

A pesar de la baja incidencia de muerte materna por COVID-19 encontrada, un estudio Sueco identificó mayor mortalidad en gestantes con COVID-19 que en no gestantes, 14,4 por 100.000 *(IC* del 95%: 7,3-23,4) y 2,5 por 100.000 (IC del 95%: 1,8-3,5), respectivamente. Cuando se incluyen sólo los casos que requirieron ventilación mecánica invasiva hubo una incidencia de 7,4 por 100.000 para las embarazadas o en posparto y de 1,8 por 100.000 para mujeres no embarazadas^26^.

Cuatro revisiones^31^^,^^44^^,^^69^^,^^92^ estuvieron de acuerdo con que la mayoría de gestantes con COVID-19 dieron a luz por cesárea. En contraste, Nayak, et al^70^ no encontraron diferencia significativa en la proporción de gestantes con y sin COVID-19 que requirieron cesárea. En cuanto al parto pretérmino, 2 artículos reportan un porcentaje de este entre el 15,2% y 21,3%^31^^,^^92^, similar al 21,8% reportado en esta revisión.

Tanto el presente estudio como tres revisiones más encontraron que el peso <2500 g en neonatos hijos de madres con COVID-19 fue del 5,3% al 7,9%^31^^,^^69^^,^^92^. Adicionalmente, el reporte de sintomatología y anomalías en las Rx de tórax se demostró en una pequeña cantidad de neonatos, lo que concuerda con dos revisiones^31^^,^^69^. Cabe resaltar que en el presente artículo se encontró que 12/15 neonatos con pruebas negativas para SARS-CoV-2 presentaron síntomas, lo que hace importante una vigilancia estrecha de los posibles síntomas de neonatos hijos de madres infectadas por SARS-COV-2, así hayan resultado negativos en la pruebas.

Se reportó baja proporción de mortalidad, ingreso a UCI y VM en neonatos, siendo coherente con otras revisiones^31^^,^^44^^,^^69^^,^^92^. Adicionalmente, esta no se asoció a COVID-19 sino a prematuridad, la cual ocurrió principalmente dado al estado materno grave, por lo que se infiere que entre peor sea el estado materno, mayor riesgo de parto pretérmino puede ocurrir y por ende, la prematuridad e ingreso a UCI será mayor.

Es de destacar que gran parte de los neonatos son internados en UCI o separados de sus madres por prevención^32^^,^^35^^,^^39^^,^^42^^,^^52^^,^^57^^,^^62^^,^^98^, por lo que se debe considerar los posibles efectos en el vínculo de la diada; se han descrito otras precauciones como el no pinzamiento tardío o no permitir la lactancia^14^^,^^62^^,^^78^^,^^80^. Diferente a otros artículos que recomendaron fomentar la lactancia y la estadía en conjunto cuando el estado materno y neonatal lo permitan, con precauciones maternas como el lavado de manos, uso de mascarilla facial y distanciamiento cuando sea posible^17^^,^^19^^,^^35^^,^^60^^,^^63^^,^^70^^,^^79^^,^^87^.

En cuanto a la transmisión materno-infantil, las pruebas positivas en neonatos se reportaron en baja cantidad, al igual que en otras revisiones^31^^,^^92^. Además, la presente revisión encontró gran variedad de pruebas en otros tejidos como tejido placentario, sangre de cordón umbilical y líquido amniótico, donde también se reportaron resultados positivos en muy baja proporción; sólo una revisión reportó un caso positivo en líquido amniótico^44^, mientras que en otras no se encontraron resultados positivos en ningún tejido^31^^,^^92^.

Teniendo en cuenta la ausencia de pruebas positivas en todas las muestras de leche materna y secreciones vaginales reportadas en la presente revisión, se considera improbable la transmisión del virus a través de la leche materna o el canal de parto, siendo consistente con otro artículo de revisión^34^. A pesar de esto, es necesario tomar precauciones para no contaminar el canal vaginal con heces maternas por un caso positivo en materia fecal.

Los resultados anteriores evidencian poca posibilidad de transmisión materno infantil si se tiene precauciones durante el parto y se imparten prácticas de autocuidado a la madre, tal como expresan otros autores^17^^,^^63^^,^^69^^,^^85^. Incluso, se plantea la posibilidad de permitir el parto vaginal con medidas preventivas^93^.

En lo referente al tratamiento de las gestantes con COVID-19, se encontró que los medicamentos más usados fueron los antibióticos seguidos de los antivirales. Por otro lado, se evidenció baja administración de hidroxicloroquina, al igual que en otro estudio de revisión^44^. Este medicamento se ha usado con precaución por la posibilidad de atravesar la barrera placentaria, su vida media extensa y sus posibles efectos secundarios neurológicos o cardíacos^50^.

Teniendo que la COVID-19 es una enfermedad reciente, solo se han descrito las complicaciones a corto plazo, por lo que es importante hacer un seguimiento a las madres con esta enfermedad y a sus hijos para determinar los efectos a largo plazo^64^, pues se ha descrito aumento de la posibilidad de enfermedades psiquiátricas en hijos de madres con infecciones durante la gestación^25^^,^^37^. Así mismo, es importante hacer un seguimiento a los hijos de mujeres que se infectaron en el primer trimestre de gestación para indagar la transmisión vertical.

## Conclusión

Las gestantes con COVID-19 suelen tener fiebre, tos y patrón de vidrio esmerilado en la TAC. La mayoría desarrollan enfermedad leve a moderada, aún así presentan más riesgo de muerte e ingreso a UCI que las no embarazadas, con aumento de la probabilidad de parto pretérmino y cesárea. Las alteraciones neonatales estuvieron mediadas principalmente por el estado materno y no se reportaron muertes perinatales asociadas a la COVID-19. Así mismo, se evidenció muy poca probabilidad de transmisión materno infantil del virus.

Además, a menos de la mitad de las gestantes se les administró algún medicamento, los más comunes fueron antibióticos y antivirales. Pero los resultados no son concluyentes por la falta de reporte de eventos adversos y la ausencia de estudios que evidencien la efectividad o no de los diferentes medicamentos utilizados. Por lo que es de vital importancia el desarrollo de más investigaciones clínicas para ampliar más la comprensión de los riesgos del virus en la gestación, los posibles efectos en el feto y en el neonato.
